# Supplementary material for: High CDC20 levels increase sensitivity of cancer cells to MPS1 inhibitors
Source: EMBO Rep. 2025 Jan 21;26(4):1036–61. doi: 10.1038/s44319-024-00363-8 (PMC11850905; doi:10.1038/s44319-024-00363-8)
Supplement: Supplementary file 1 — Table EV1 [file 44319_2024_363_MOESM1_ESM.docx]

**Table EV1**. Lists of genes included in the APC/C signatures analyzed in Fig. 2.

**Subunit only APC/C signature:**

CDC27, ANAPC4, ANAPC5, CDC23, ANAPC15, ANAPC13, CDC16, ANAPC11, ANAPC1, ANAPC10, ANAPC16, ANAPC2, ANAPC7, CDC26

**Extended APC/C signature (used by Thu et al, PNAS, 2018):**

CDC27, ANAPC4, ANAPC5, CDC23, ANAPC15, ΑΝΑPC13, CDC16, ANAPC11, ANAPC1, ANAPC10, ANAPC16, ANAPC2, ANAPC7, CDC26, MAD2L1BP, UBE2C, CDC20
