# Supplementary material for: High CDC20 levels increase sensitivity of cancer cells to MPS1 inhibitors
Source: EMBO Rep. 2025 Jan 21;26(4):1036–61. doi: 10.1038/s44319-024-00363-8 (PMC11850905; doi:10.1038/s44319-024-00363-8)
Supplement: Supplementary file 2 — Table EV2 [file 44319_2024_363_MOESM2_ESM.docx]

**Table EV2**. shRNA and qPCR primer sequence data

shRNA:

| species | gene | sequence |
| --- | --- | --- |
| mouse | Cdc20 shRNA1 | GCAGCAGAAACGACTTCGAAA |
|  | Cdc20 shRNA2 | GCCGAACTCCTGGCAAATCTA |
| Human | CDC20 shRNA1 | AGACCAACCCATCAC |
|  | CDC20 shRNA2 | CCCATTACAAGGAGCTCAT |

Primer sequences used for quantitative real-time PCR (qRT-PCR):

| species | gene | 5' to 3' sequence | primer |
| --- | --- | --- | --- |
| mouse | Cdc20 | Sense | TTCGTGTTCGAGAGCGATTTG |
|  | Cdc20 | Anti-sense | ACCTTGGAACTAGATTTGCCAG |
|  | Actb | Sense | CTAGGCACCAGGGTGTGATG |
|  | Actb | Anti-sense | GGCCTCGTCACCCACATAG |
| human | CDC20 | Sense | GACCACTCCTAGCAAACCTGG |
|  | CDC20 | Anti-sense | GGGCGTCTGGCTGTTTTCA |
|  | Tubulin | Sense | CTTCGTCTCCGCCATCAG |
|  | Tubulin | Anti-sense | CGTGTTCCAGGCAGTAGAGC |
