# Supplementary material for: High CDC20 levels increase sensitivity of cancer cells to MPS1 inhibitors
Source: EMBO Rep. 2025 Jan 21;26(4):1036–61. doi: 10.1038/s44319-024-00363-8 (PMC11850905; doi:10.1038/s44319-024-00363-8)
Supplement: Supplementary file 28 — Expanded View Figures [file 44319_2024_363_MOESM28_ESM.pdf]

Expanded View Figures

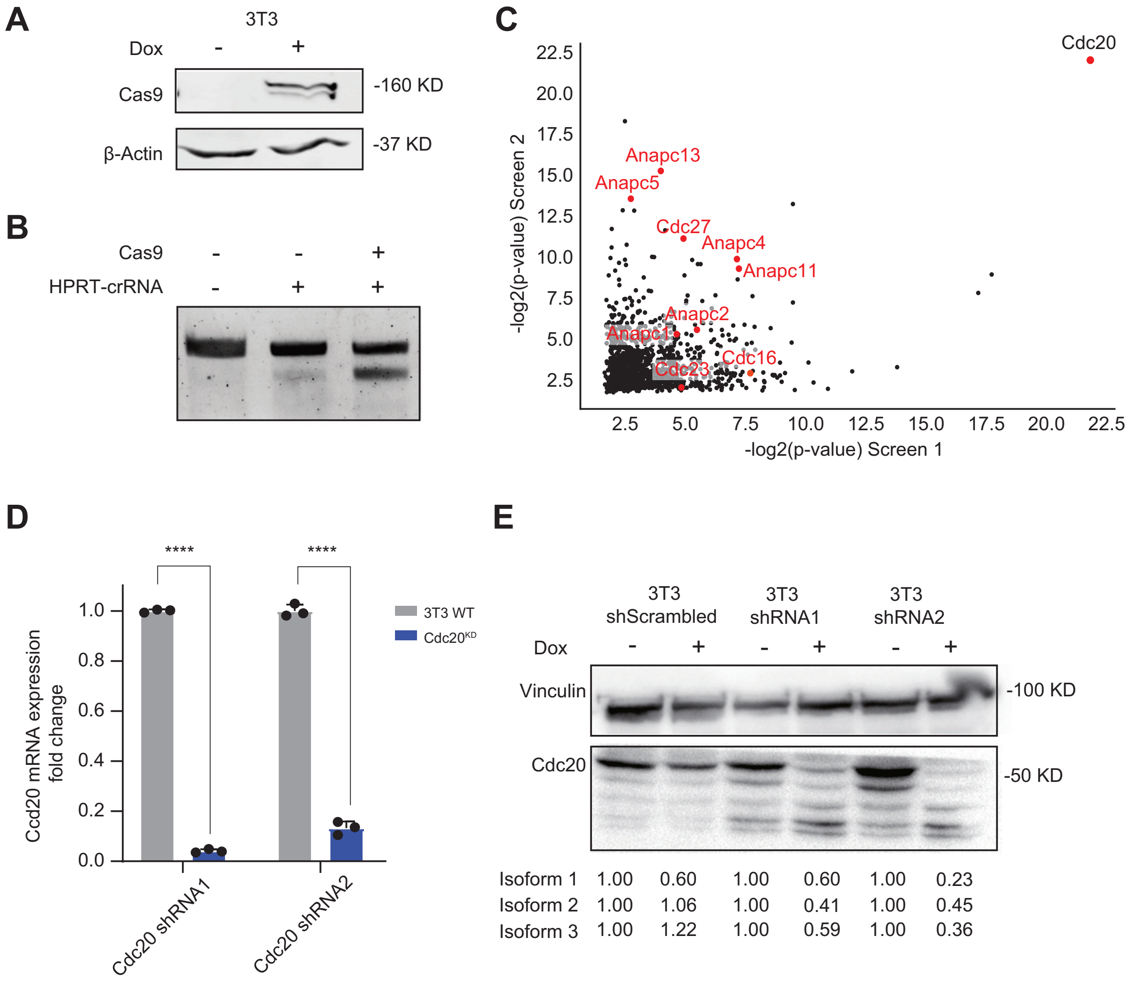

Figure EV1. CDC20 is strongly associated with resistance to SAC inhibition.

(**A**) Western blot validation for Cas9 expression in 3T3 cells used for the CRISPR screen. (**B**) Cleaved and uncleaved PCR product in a T7 assay as a readout of Cas9 activity in 3T3 cells used for the CRISPR screen. (**C**) Correlation between the top-ranked 25% of genes in both CRISPR screens based on their statistical significance with all APC/C-related genes highlighted, showing that Cdc20 is by far the most significant outlier of all APC/C extended complex members. *P* values were calculated using the RRA method (see "Methods"). (**D**, **E**) qPCR (**D**) or western blot (**E**) validation of Cdc20 knockdown by shRNA. Paired *t* test (*N*, number of biological replicates; $N = 3$; ****, P value < 0.0001). Error bars represent the standard deviation (SD) of the mean. Source data are available online for this figure.

© The Author(s)

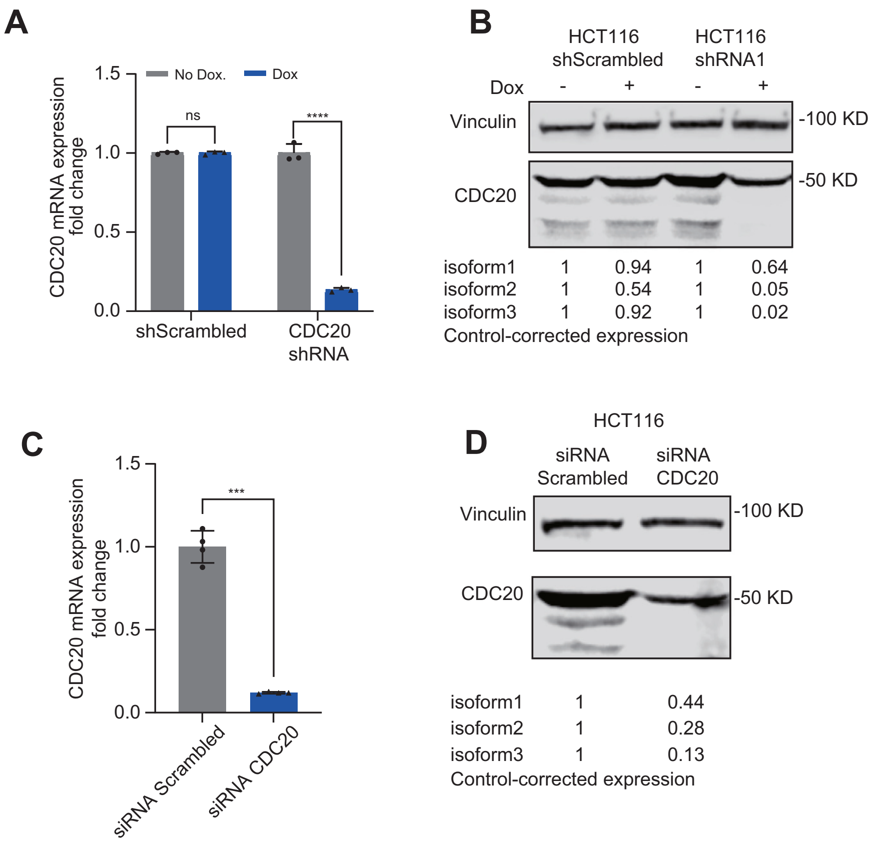

Figure EV2. CDC20 expression predicts sensitivity to genetic and chemical SAC perturbation.

(**A**, **B**) qPCR (**A**) and western blot (**B**) validation of CDC20 knockdown by shRNA in HCT116 cells. Two-sided paired *t* test (*N*, number of biological replicates; $N = 3$; ns, *P* value = 0.9999; ****, *P* value < 0.0001). Error bars represent the standard deviation (SD) of the mean. (**C**, **D**) qPCR (**C**) and western blot (**D**) validation of CDC20 knockdown by siRNA in HCT116 cells. Two-sided paired *t* test ($N = 4$; ***, *P* value = 0.001). A representative image is shown. Quantification values of the individual bands show the average value of all replicates. Source data are available online for this figure.

 © The Author(s)

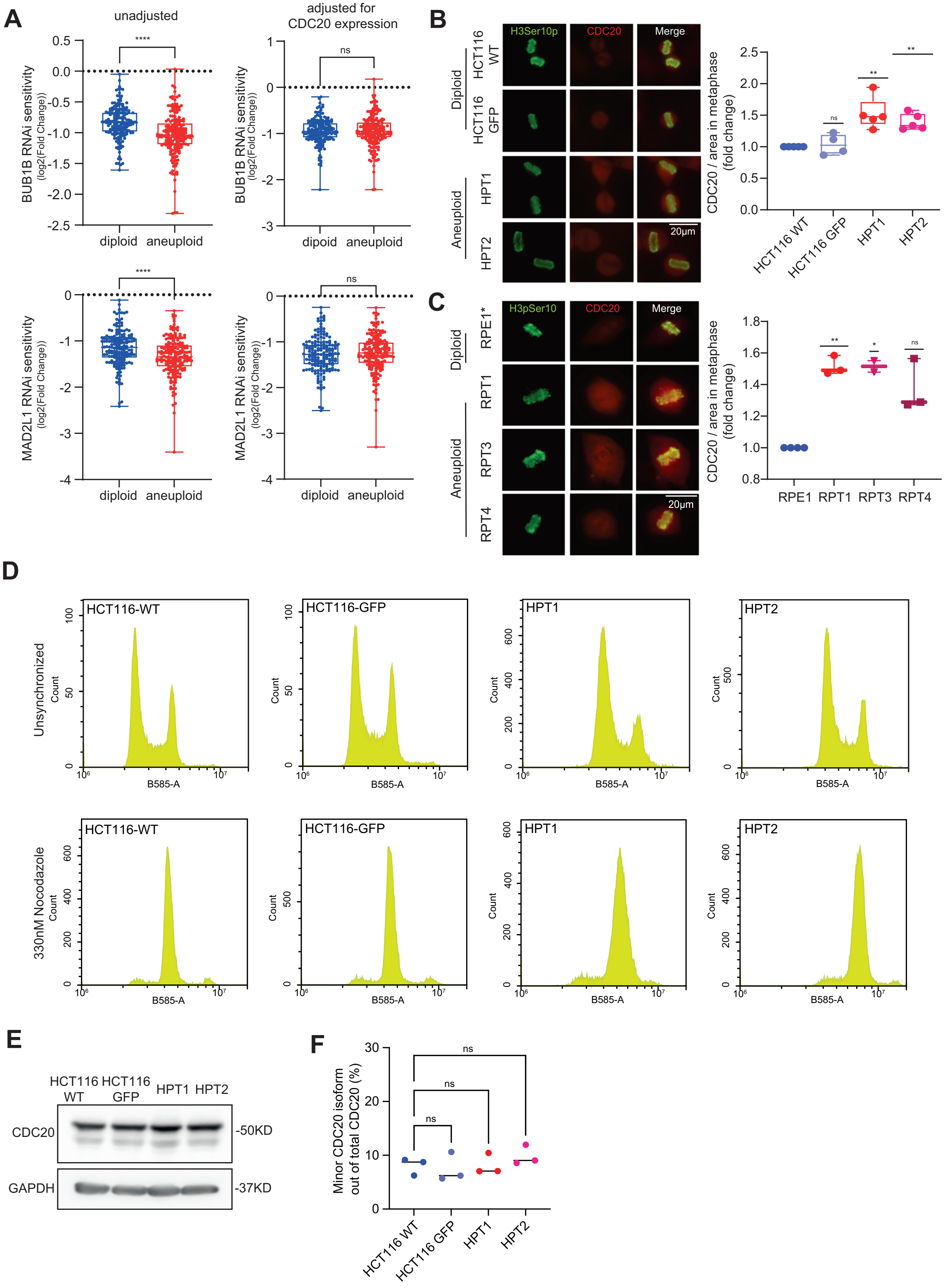
A
unadjusted
adjusted for
CDC20 expression
BUB1B RNAi sensitivity
(log2(Fold Change))
MAD2L1 RNAi sensitivity
(log2(Fold Change))
****
ns
diploid aneuploid
dipoid aneuploid
B
H3Ser10p
CDC20
Merge
Diploid
HCT116 WT
HCT116 GFP
Aneuploid
HPT1
HPT2
20μm
CDC20 / area in metaphase
(fold change)
HCT116 WT
HCT116 GFP
HPT1
HPT2
C
H3pSer10
RPE1*
RPT1
RPT3
RPT4
RPE1 RPT1 RPT3 RPT4
D
HCT116-WT
HCT116-GFP
HPT1
HPT2
Unsynchronized
330nM Nocodazole
Count
B585-A
E
HCT116 WT
HCT116 GFP
HPT1 HPT2
CDC20
GAPDH
-50KD
-37KD
F
Minor CDC20 isoform
out of total CDC20 (%)
ns

Figure EV3. Increased CDC20 expression is associated with the preferential response of aneuploid cells to SAC inhibition.

(**A**) Left - correlation between ploidy and the sensitivity to genetic perturbation of the core SAC components BUB1B (top) and MAD2L1 (bottom). Right – same correlation with CDC20 expression removed as a linear covariate (see "Methods"). When removing the effect of CDC20 expression the trend reverses or becomes insignificant. Box plots show the median (middle line) and interquartile range (IQR; box edges). Whiskers extend to values within 1.5×IQR from the 25th and 75th percentiles. Two-sided *t* test (ns, P value = 0.5302 for MAD2L1 RNAi and 0.8019 for BUB1B RNAi; ****, P value < 0.0001). (**B**, **C**) Representative images (left) and single-cell quantification (right) of CDC20 at metaphase in cells of the HCT116-HPT system (**B**) or the RPE-RPT system (**C**) after synchronization with 9 nM or 4.5 nM RO-3306 (respectively) for 20 h. Highly aneuploid cells express higher levels of CDC20 than their diploid counterparts. In (**B**), box plots show the median (middle line) and interquartile range (IQR; box edges). Whiskers extend to values within 1.5×IQR from the 25th and 75th percentiles. In (**C**), bars represent the data range. One-sample *t* test (N, number of biological replicates; $N = 5$ or $N = 4$ respectively; ns, P value = 0.7017 (**B**) or P value 0.0583 (**C**); *, P value = 0.0447, **, P value = 0.0093 or P value = 0.0023 (**B**, left to right) and P value = 0.0046 (**C**)). (**D**) Flow cytometry analysis showing the cell cycle distribution of HCT116-HPT cells in an unsynchronized state (top) or after synchronization with 330 nM Nocodazole for 20 h (bottom), the same conditions that were used for the bulk CDC20 quantification in Fig. 3D. All four cell lines are synchronized to a similar extent, allowing for a bulk comparison of a cell cycle protein expression. (**E**) Representative western blot image of bulk CDC20 expression in the HCT116-HPT cell line set following synchronization with 330 nM Nocodazole. Aneuploid cells express higher levels of CDC20 than their diploid counterparts. (**F**) Percent of minor CDC20 isoform out of total CDC20, as observed in bulk quantification. There is no significant difference in the fraction of minor isoform between the diploid and aneuploid cell lines. One-way ANOVA (N, number of biological repeats; $N = 3$; ns, P value = 0.9781 or P value = 0.9996 or P value = 0.5945 (from left to right)). Source data are available online for this figure.

© The Author(s)

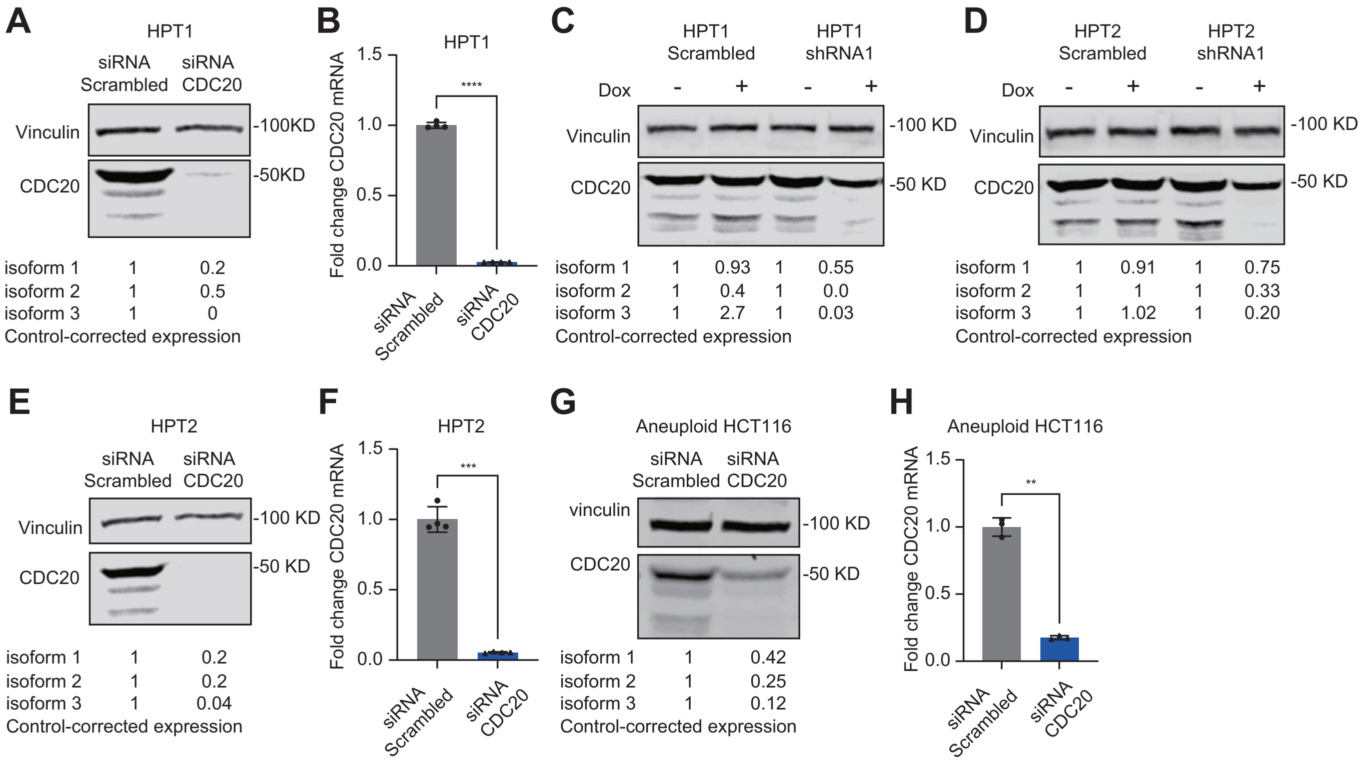

Figure EV4. Validation of CDC20 knockdown in aneuploid cell lines.

(**A**, **B**) Western blot (**A**) and qPCR (**B**) validation of CDC20 knockdown by siRNA in HPT1 cells. Two-sided *t* test (*N*, number of biological replicates; $N = 4$; ****, *P* value < 0.0001). Error bars represent the standard deviation (SD) of the mean. A representative image is shown; Quantification values of the individual bands show the average value of all replicates. (**C**, **D**) Western blot validation of CDC20 depletion by shRNA in HPT1 (**C**) and HPT2 (**D**). (**E**, **F**) Western blot (**E**) and qPCR (**F**) validation of CDC20 knockdown by siRNA in HPT2 cells. Two-sided *t* test (*N*, number of biological repeats; $N = 4$; ***, *P* value = 0.0002). (**G**, **H**) Western blot (**G**) and qPCR (**H**) validation of CDC20 knockdown by siRNA in aneuploid HCT116 cells. Two-sided *t* test ($N = 3$; **, *P* value = 0.0034). Source data are available online for this figure.

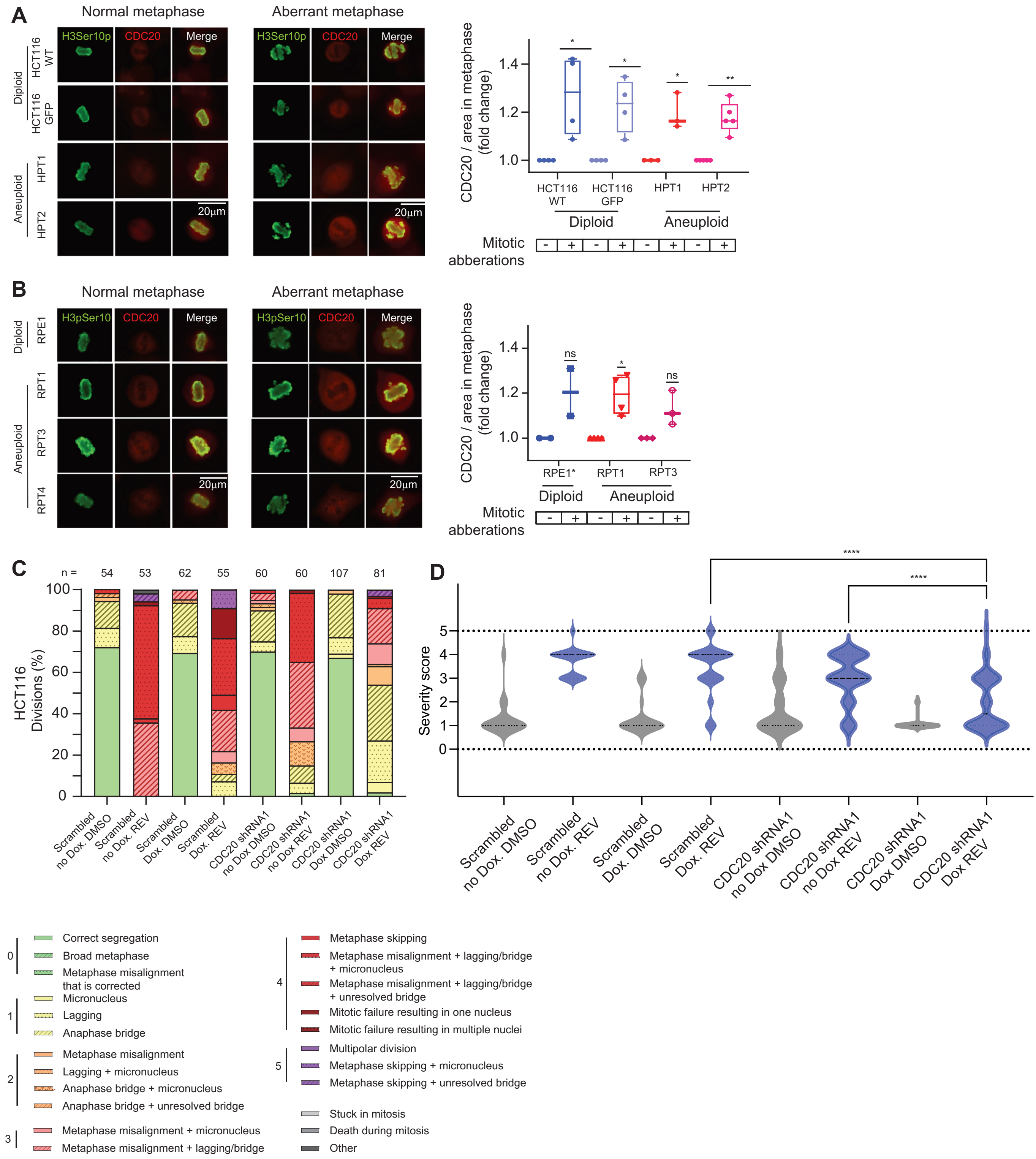
A
Normal metaphase
Aberrant metaphase
H3Ser10p
CDC20
Merge
Diploid
HCT116 WT
HCT116 GFP
Aneuploid
HPT1
HPT2
20µm
CDC20 / area in metaphase (fold change)
HCT116 WT
HCT116 GFP
HPT1
HPT2
Diploid
Aneuploid
Mitotic abberations
B
H3pSer10
RPE1
RPT1
RPT3
RPT4
RPE1*
ns
C
HCT116 Divisions (%)
n = 54 53 62 55 60 60 107 81
Scrambled no Dox. DMSO
Scrambled no Dox. REV
Scrambled Dox. DMSO
Scrambled Dox. REV
CDC20 shRNA1 no Dox DMSO
CDC20 shRNA1 no Dox REV
CDC20 shRNA1 Dox DMSO
CDC20 shRNA1 Dox REV
D
Severity score
Correct segregation
Broad metaphase
Metaphase misalignment that is corrected
Micronucleus
Lagging
Anaphase bridge
Metaphase misalignment
Lagging + micronucleus
Anaphase bridge + micronucleus
Anaphase bridge + unresolved bridge
Metaphase misalignment + micronucleus
Metaphase misalignment + lagging/bridge
Metaphase skipping
Metaphase misalignment + lagging/bridge + micronucleus
Metaphase misalignment + lagging/bridge + unresolved bridge
Mitotic failure resulting in one nucleus
Mitotic failure resulting in multiple nuclei
Multipolar division
Metaphase skipping + micronucleus
Metaphase skipping + unresolved bridge
Stuck in mitosis
Death during mitosis
Other

 © The Author(s)

Figure EV5. CDC20 expression levels determine the prevalence of mitotic errors and metaphase duration.

(**A**, **B**) Representative IF images (left) and quantification (right) of CDC20 in HCT116-HPT cells (**A**) or RPE-RPT cells (**B**) undergoing normal or aberrant mitoses after synchronization with RO-3306. Cells with mitotic aberrations express significantly higher levels of CDC20 during metaphase than cells undergoing normal division, regardless of ploidy background. In (**A**), Box plots show the median (middle line) and interquartile range (IQR; box edges). Whiskers extend to values within 1.5×IQR from the 25th and 75th percentiles. In (**B**), bars represent the data range. One-sample *t* test (*N*, number of biological replicates; $N = 5$ (A) and $N = 4$ (B); ns, *P* value = 0.3037 or 0.1028 (**B**, from left to right); *, *P* value = 0.0498 or *P* value = 0.0271 or *P* value = 0.0465 (**A**, left to right) and *P* value = 0.0233 (**B**); **, *P* value = 0.0033). (**C**, **D**) Distribution (**C**) and severity quantification (**D**) of mitotic abnormalities in HCT116 cells treated with 125 nM Reversine, under control conditions or CDC20 depletion by shRNA. Mitotic aberrations were identified by live-cell imaging and scored on a severity scale of 0–5, then grouped and colored by score. The changes in severity distribution across samples were assessed using one-sided Kruskal–Wallis tests, as elaborated in "Methods" section. Cells treated with Reversine exhibit reduced mitotic aberrations after CDC20 depletion. Sample size (*N*) in (**D**) corresponds to the sample size in (**C**). One-sided Kruskal–Wallis test (****, *P* value < 0.0001). Source data are available online for this figure.

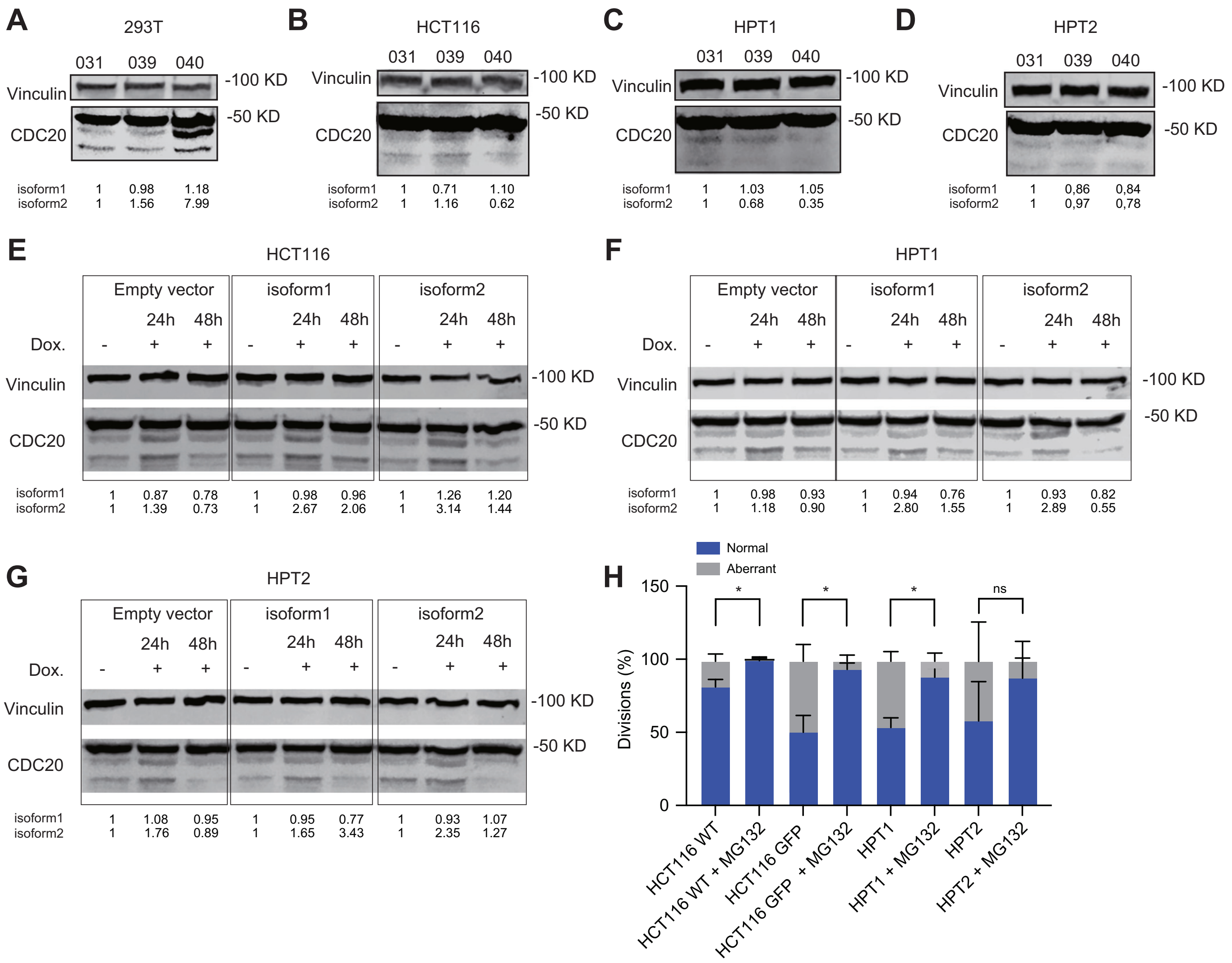

Figure EV6. CDC20 overexpression attempts in HCT116-HPT cells.

(**A**) Western blot quantification of CDC20 overexpression with plasmids from Tsang et al (Tsang and Cheeseman, 2023), in 293T cells. CDC20 is overexpressed in these cells. (**B–D**) Western blot quantification of CDC20 overexpression with plasmids from Tsang et al 2023, in HCT116 (**B**), HPT1 (**C**) and HPT2 (**D**) cells. No CDC20 overexpression can be detected in these cells. (**E–G**) Western blot quantification of CDC20 overexpression with lentiviral rTTA inducible system, in HCT116 (**E**), HPT1 (**F**) and HPT2 (**G**) cells. No CDC20 overexpression can be detected in this system either. (**H**) Rate of normal and abnormal cell divisions in the HCT-HPT system during normal (45 min) and prolonged (120 min) metaphases (see legend for Fig. 5G, "Methods"). The rate of mitotic aberrations is significantly decreased in cells undergoing prolonged metaphases. Two-sided paired *t* test (*N*, number of biological replicates; $N = 3$; ns, *P* value = 0.0929; *, *P* value = 0.0251 or *P* value = 0.0385 or *P* value = 0.0210 (from left to right)). Source data are available online for this figure.

 © The Author(s)
