# Supplementary material for: High CDC20 levels increase sensitivity of cancer cells to MPS1 inhibitors
Source: EMBO Rep. 2025 Jan 21;26(4):1036–61. doi: 10.1038/s44319-024-00363-8 (PMC11850905; doi:10.1038/s44319-024-00363-8)
Supplement: Supplementary file 27 — EV Figure Source Data [file 44319_2024_363_MOESM27_ESM.zip › Figure_EV1.zip!/Figure_EV1/Figure_EV1B/Figure_EV1B.pdf]

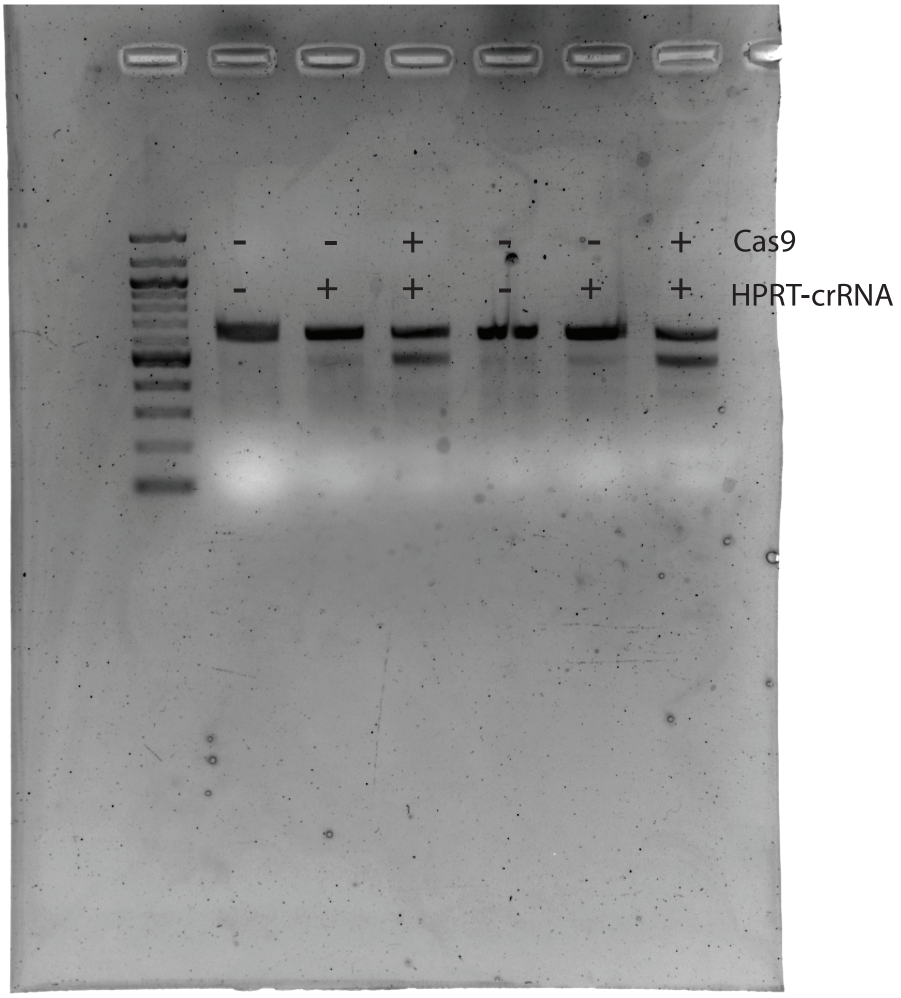
- - + - - + Cas9
- + + - + + HPRT-crRNA
